# Selective Inhibitor of the c-Met Receptor Tyrosine Kinase in Advanced Hepatocellular Carcinoma: No Beneficial Effect With the Use of Tivantinib?

**DOI:** 10.3389/fimmu.2021.731527

**Published:** 2021-11-02

**Authors:** Shankun Zhao, Weizhou Wu, Hao Jiang, Lei Ma, Chengyi Pan, Chong Jin, Jinggang Mo, Liezhi Wang, Kunpeng Wang

**Affiliations:** ^1^ Department of Urology, Taizhou Central Hospital (Taizhou University Hospital), Taizhou, China; ^2^ Department of Urology, Maoming People’s Hospital, Maoming, China; ^3^ Department of General Surgery, Taizhou Central Hospital (Taizhou University Hospital), Taizhou, China

**Keywords:** tivantinib, MET inhibitor, hepatocellular carcinoma, therapeutic effect, adverse event

## Abstract

Advanced hepatocellular carcinoma (HCC) remains a formidable health challenge worldwide, with a 5-year survival rate of 2.4% in patients with distant metastases. The hepatocyte growth factor/cellular-mesenchymal-epithelial transition (HGF/c-Met) signaling pathway represents an encouraging therapeutic target for progressive HCC. Tivantinib, a non-adenosine triphosphate-competitive c-Met inhibitor, showed an attractive therapeutic effect on advanced HCC patients with high MET-expression in phase 2 study but failed to meet its primary endpoint of prolonging the overall survival (OS) in two phase 3 HCC clinical trials. Seven clinical trials have been registered in the “ClinicalTrials.gov” for investigating the safety and efficacy of tivantinib in treating advanced or unresectable HCC. Eight relevant studies have been published with results. The sample size ranged from 20 to 340 patients. The methods of tivantinib administration and dosage were orally 120/240/360 mg twice daily. MET overexpression was recorded at 34.6% to 100%. Two large sample phase 3 studies (the METIV-HCC study of Australia and European population and the JET-HCC study of the Japanese population) revealed that tivantinib failed to show survival benefits in advanced HCC. Common adverse events with tivantinib treatment include neutropenia, ascites, rash, and anemia, etc. Several factors may contribute to the inconsistency between the phase 2 and phase 3 studies of tivantinib, including the sample size, drug dosing, study design, and the rate of MET-High. In the future, high selective MET inhibitors combined with a biomarker-driven patient selection may provide a potentially viable therapeutic strategy for patients with advanced HCC.

## Background

Liver cancer remains a major global health challenge. According to Global Cancer Statistics 2020 (1), primary liver cancer ranges the 6th most frequently diagnosed malignancy and the 3rd leading cause of cancer death worldwide in 2020. Hepatocellular carcinoma (HCC), the most common type of primary liver cancer, comprises 75%-85% of cases and has an increasing incidence worldwide (1). The 5-year survival rate in HCC patients with distant metastases is only 2.4%. The pathophysiology of HCC is a complex multistep developmental process that interplays with various factors, including genetic predisposition, the cellular microenvironment, and various immune cells, etc (2).

In the management of HCC, resection, transplantation, and local ablation are the preferred candidates for the early-stage sufferers (3). Transarterial chemoembolization (TACE) is recommended for patients at intermediate stages, while those with advanced disease are suggested to firstly receive systemic therapies (3). Systemic therapies are one of the choices for managing advanced or unresectable HCC. Sorafenib is a first-line systemic treatment for unresectable HCC (4). Several new effective drugs, i.e., lenvatinib, regorafenib, cabozantinib, ramucirumab, nivolumab, and pembrolizumab have been established for the systemic treatment of HCC since 2017 (5). According to the positive data of the phase III trials, three regimens [regorafenib (6), cabozantinib (7), and ramucirumab (8)] are approved for the treatment of patients with advanced HCC who had failed to sorafenib therapy.

Tivantinib is a selective small molecular inhibitor of cellular mesenchymal-epithelial transcription factor (c-Met or MET) that was clinically developed in various cancers, including non-small-cell lung cancer (9), colon cancer (10), gastric cancer (11), and HCC (12). c-Met (historically identified as the product of human MET gene), a receptor tyrosine kinase, binds its sole ligand hepatocyte growth factor (HGF), which activates intracellular signaling pathways, such as RAS-mitogen activated protein kinase (MAPK), MEK, PI3K-AKT, STAT3, beta-catenin, and Notch pathway (13, 14). HGF is the unique c-Met ligand that dramatically stimulates c-Met activation, acting by Des-γ-carboxy-prothrombin or cell attachment independent of the ligand, to alter epithelial-mesenchymal interactions (15, 16). Aberrant HGF/c-Met signaling activation has been identified in multiple cancers. The binding of HGF to c-Met’s Sema domain results in receptor homodimerization, autophosphorylation of tyrosine residues in the tyrosine kinase domain, and downstream activation of some signaling pathways, which contribute to the malignant transformation of a wide range of cancers (17, 18). Thus, tivantinib exhibits its potent antitumor activity by inhibiting HGF/c-Met signaling activation. The binding of the hepatocyte growth factor/scatter factor (HGF/SF) to MET activates the involving signaling pathways, leading to cell growth, migration, invasion, and resistance to apoptosis (12, 19). The non-canonical activation of c-Met triggers carcinogenesis by the molecular variants of MET, such as amplification and splicing (20). In addition, hyperactive HGF/c-Met signaling also promotes the growth, survival, and metastasis of the cancer cells (21). As reported, c-Met plays a tumor-promoting function in cancer largely mediated through fostering metabolism reprogramming of cancer cells and reinforcing the cancer stemness (22, 23). Clinically, a high level of c-MET is negatively correlated with the prognosis of multiple cancers, i.e., lung cancer (24), breast cancer (25), and clear cell renal cell carcinoma (26), etc. Numerous studies have suggested that there is a positive relationship between HGF/c-Met signaling and the proliferation, regeneration, and survival of HCC (27). Mechanistically, elevated c-Met transcription and overexpression of c-Met are associated with dysregulation of downstream signaling cascades (i.e., STAT3, MEK, and PI3K-AKT), MET missense mutations and MET splicing, promoting HCC cell metabolism and biogenesis, or driving tumor-initiating stem-like cell phenotype (28–30). Aberration of the proto-oncogene c-Met is identified on nearly half of the HCC patients and all liver metastases (31). Teufel et al. even found that the plasma level of c-Met was 100% increased, which was correlated with a worse prognosis in patients with HCC (32). In line with this finding, Asaoka et al. also observed that a high level of c-Met as a promising prognostic factor for HCC (33). Therefore, c-Met blockade therapy might have the function of suppressing HCC development. Presumably due to MET inhibition, tivantinib has been speculated to have antiproliferative effects on HCC cells.

Currently, several phase I/II/IIIW clinical trials on tivantinib, a selective oral inhibitor of c-MET, have been completed. The therapeutic effects were controversial among these clinical trials. In this review, we aimed to summarize all the clinical evidence investigating tivantinib on advanced-stage HCC, which might facilitate the clinical understanding of the treatment efficacy and adverse reactions of tivantinib in HCC.

### Overview of Tivantinib

**Figure 1:**
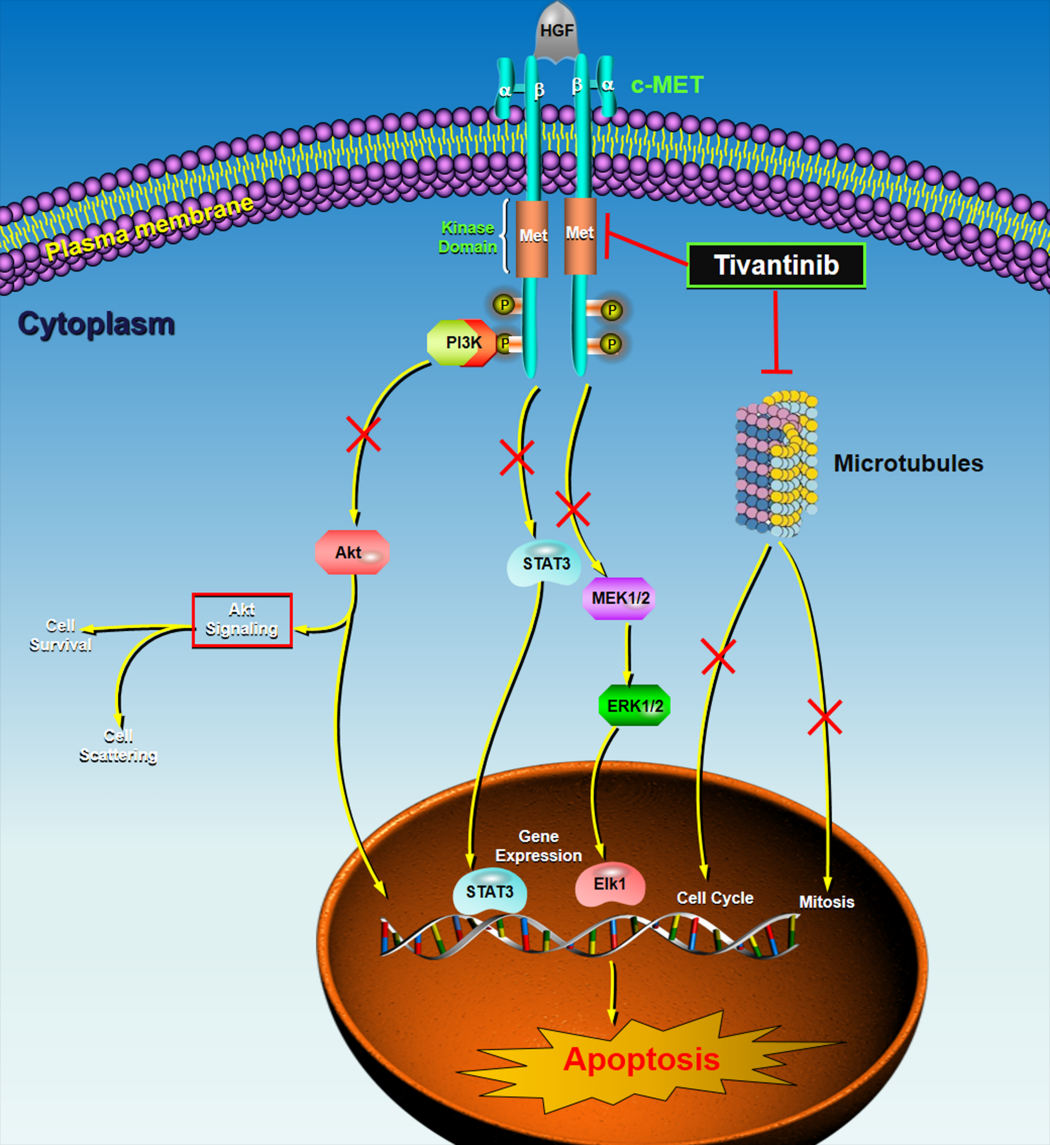
The schematic diagram of the molecular mechanisms of tivantinib in treating HCC. c-MET is the high-affinity receptor for the HGF. c-MET is a single-chain precursor protein composed of extracellular α-subunit and a transmembrane β-subunit. The HGF/c-MET axis commonly dysregulates in cancers, including HCC. Tivantinib, a small molecule c-MET inhibitor, targets the inactive, unphosphorylated form of c-MET, locking it in the inactive configuration and suppressing downstream intracellular signaling pathways, such as PI3K-AKT, STAT3, and MEK-ERK. Also, tivantinib can directly bind microtubules, inducing mitotic catastrophe and cell cycle arrest by disrupting microtubule function or microtubule depolymerization. These actions driving by tivantinib independently or collectively contribute to subsequent apoptosis of the cancer cells. HGF, hepatocyte growth factor; MET, mesenchymal-epithelial transcription factor; PI3K, phosphoinositide 3-kinase; STAT, signal transducer and activator of transcription; MEK, mitogen-activated protein kinase kinase; ERK, extracellular signal-regulated kinase; P, phosphorylation.

Tivantinib, formerly known as ARQ 197, is an orally administered, highly selective (10–100-times more selective for MET than 229 other kinases tested), non-ATP competitive inhibitor of c-MET, with an inhibitory constant (Ki) of 355 nmol (34). Tivantinib is a totally synthetic small molecule with the bis-substituted pyrrolidine-2, 5-dione structure. Its chemical formula is (3R,4R)-3-(5,6-dihydro-4H-pyrrolo[3,2,1-ij]quinolin-1-yl)-4-(1Hindol-3-yl)pyrrolidine-2,5-dione (35). Tivantinib had an elimination half-life of 29 min in human microsomes, metabolized by CYP 2C19 and CYP 3A4, with IC_50_ values >10 μM (12). Tivantinib strongly inhibits MET autoactivation by stabilizing the inactive nonphosphorylated configuration of the kinase, leading to the arrest of cell growth (36). It arrests MET-dependent downstream signaling pathways by perturbing constitutive and ligand-mediated MET phosphorylation (34), resulting in a reduction in proliferation, invasion, metastasis, and the induction of caspase-dependent apoptosis (37). Mechanically, tivantinib distinguishes itself from other c-Met inhibitors due to it disrupts c-Met phosphorylation in a non-ATP competitive manner (38), despite some investigators failed to find that tivantinib can suppress c-MET phosphorylation (39, 40). Clinically, circulating MET expression serves as a pharmacodynamic biomarker for predicting tivantinib efficacy in advanced HCC (41). A randomized, phase 2 trial indicated that MET might be an independent prognostic factor for overall survival in advanced HCC (42). This study showed that patients with MET-high tumors had significantly shorter survival compared with the MET-low subgroup. As for another key factor of HGF/c-Met signaling, some investigators found that the high levels of circulating HGF might be an independent poor prognostic factor in patients with advanced cancers (43). α-Fetoprotein (AFP) is the serum biomarker most widely used in HCC, but it did not significantly contribute to predicting prognosis and monitor response to tivantinib therapy (41). Other mechanisms of action for tivantinib might be due to it could directly bind microtubules, thus disrupting microtubule function inducing mitotic catastrophe, and leads to subsequent apoptosis (39, 44). Tivantinib could inhibit cell viability regardless of MET activation in cancer cells, thus microtubule inhibition might be the key mechanism of tivantinib-associated HCC growth inhibition (45). **Figure 1** displays the schematic diagram of the molecular mechanisms of tivantinib in treating HCC.

### *In Vitro* and *In Vivo* Studies of Tivantinib

Several *in vitro* and *in vivo* studies have been conducted for investigating the biomedical function of tivantinib in multiple cancers. The results are promising. In an *in vitro* study (46), Munshi et al. demonstrated that ARQ 197 has broad-spectrum antineoplastic effects, including lung cancer, melanoma, breast cancer, colon cancer, ovarian cancer, and gastric cancer, by inhibiting the downstream c-Met effectors and in turn exhibiting the antiproliferative and proapoptotic effects. Munshi et al. also conducted an *in vivo* study and found that oral administration of tivantinib with 200 mg/kg significantly reduced levels of phosphorylated MET in xenograft tumors and decreased the tumor volumes (46). Previdi et al. (47) explored the pharmacological effects of tivantinib by using a specific short hairpin RNA against the c-Met mouse model of breast cancer. They found that a 120 mg/kg dose of tivantinib could dramatically repress the growth of the subcutaneous tumor, metastatic growth of breast cancer cells in bone, and tumor-induced osteolysis. Calles et al. (40) indicated that tivantinib was associated with a G2/M arrest and induced apoptosis in non-small-cell lung cancer (NSCLC) cell lines.

There are six *in vitro*/vivo studies that have investigated the role of tivantinib in HCC. Xiang et al. (48) demonstrated that tivantinib administration could induce G2/M arrest and promote apoptosis by disrupting tubulin polymerization, exhibited an anti-tumor growth activity in HCC. An *in vitro* study developed by Lu et al. (49) revealed that Bcl-xl, Mcl-1, and Cyclin B1 served as the mediators of the anti-tumor effects of tivantinib in HCC and suggested that these molecules might be the reliable markers for patients’ selection. Rebouissou et al. (50) confirmed that tivantinib acted as an antimitotic compound for the treatment of HCC by conducting an *in vitro* study. They also found that cell proliferation markers, i.e., Ki67, might be a candidate predictor to evaluate the antitumor efficacy of tivantinib. Gao et al. (51) found that treatment with tivantinib remarkably enhanced the sensitivity of HCC cells to sorafenib and indicated that the underlying mechanism might be correlated to suppress the expression of EMT- and MDR-related genes. The authors further observed that tivantinib decelerated the clearance of sorafenib in HCC cells and subcutaneous HCC tumors in mice, showing the enhancement of the antitumor effect of sorafenib. Kobayashi et al. (52) reported that tivantinib regulated BCRP upstream of exon 1α in HCC HepG2 cells and suggested that tivantinib could be administrated in combination therapy with 5-FU as hepatic arterial infusion chemotherapy (HAIC) and sorafenib against HCC. In a more recent study, Rashed et al. (53) showed that the response to tivantinib in HCC cell lines was dramatically associated with MET RNA expressions but not L1-MET or MET protein expressions. As a result, the level of MET RNA might be a useful biomarker for tivantinib targeted therapy in HCC.

Taken together, based on the experimental data, tivantinib, a non-ATP competitive inhibitor of c-Met, exhibits a promising anti-tumor effect in HCC therapy. Mechanistic studies suggested that the antineoplastic function of tivantinib might be correlated with its anti-proliferative and pro-apoptotic effects and its interaction with multiple affected proteins and signaling pathways.

## Clinical Studies

### Registered Studies in the ClinicalTrials

In the web of ClinicalTrials.gov (https://clinicaltrials.gov/), there are seven clinical studies have been registered for investigating the safety and the efficacy of tivantinib in treating advanced or unresectable HCC. All of these trials have been completed. The details of these clinical studies were listed in **Table 1**. Among the seven studies, four are Phase 1 trials, one is Phase 2 trial, and two are Phase 3 trials. For the study areas, three are international multicenter, two in Japan, and one in the USA. The cancer types are cirrhotic HCC, advanced HCC, MET-high HCC, and unresectable HCC. The number of patients in these studies ranged from 21 to 386. The age of the participants reports at 13, 18, or 20 years and older. The methods of administration include dosage of 120/240/360 mg tablets, administered by mouth twice daily (BID), with or after meals. The time frame ranges from 20 months to ten years. The responsible party includes Merck Sharp and Dohme and Kyowa Kirin. Only one clinical trial has provided the outcomes, that is the “NCT01755767”, which showed the median overall survival (OS) of 8.4 (6.8 to 10.0) months and 9.1 (7.3 to 10.4) months for tivantinib and placebo, respectively. The median progression-free survival in this study was reported at 2.1 months and 2.0 months in the tivantinib group and placebo group, respectively. Serious adverse events have been shown in two clinical trials, including NCT01755767 and NCT01178411. In the former one, serious adverse events in the 240 mg and 120 mg tivantinib group were recorded at 60.71% and 45.78%. In the latter one, serious adverse events were found at 31.67% with the use of tivantinib. Of note, some of these seven clinical studies have been published.

**Table 1:** Trials of tivantinib in treating HCC registered in the - ClinicalTrials.gov.

Clinical Trials ID	Study area	Status	Cancer type, Number of patients	Age (years)	Therapies (Tivantinib)	Time Frame	Responsible Party	Outcomes	Serious Adverse Events (%)
NCT00802555, Phase 1	Multi-center	Completed	Cirrhotic patients with HCC, 21	Over 18	360 mg, BID, Orally	January 2009 to December 2011	Merck Sharp and Dohme	No Results Posted	No Results Posted
NCT00827177, Phase 1	Multi-center	Completed	HCC and other solid tumors, 87	Over 18	360 mg *vs* 240 mg, BID, Orally	September 2009 to May 2013	Merck Sharp and Dohme	No Results Posted	No Results Posted
NCT01656265, Phase 1	Japan	Completed	Advanced HCC, 24	Over 20	Daily repeating dose of oral Tivantinib (lack of dose), BID, Orally	July 2012 to March 2014	Kyowa Kirin	No Results Posted	No Results Posted
NCT01755767, Phase 3	Multi-center	Completed	MET-high HCC, 383	Over 18	120 mg *vs* 240 mg *vs* Placebo, BID, Orally	December 27, 2012 to July 31, 2017	Merck Sharp and Dohme	Median OS: 8.4 (6.8 to 10.0) for Tivantinib *vs* 9.1 (7.3 to 10.4) for Placebo; Median PFS: 2.1 for Tivantinib and 2.0 for Placebo	Tivantinib 240 mg: 17/28 (60.71%); Tivantinib 120 mg: 103/225 (45.78%)
NCT00988741, Phase 2	USA	Completed	Unresectable HCC, 107	Over 18	360 mg *vs* 240 mg *vs* Placebo, BID, Orally	September 2009 to March 2012	Merck Sharp and Dohme	No Results Posted	No Results Posted
NCT02029157, Phase 3	Japan	Completed	MET-high HCC, 386	Over 20	NA	January 2014 to August 2017	Kyowa Kirin	No Results Posted	No Results Posted
NCT01178411, Phase 1	NA	Completed	HCC and other solid tumors, 60	Over 13	360 mg, BID, Orally	August 31, 2010 to January 14, 2019	Merck Sharp and Dohme	NA	19/60 (31.67%)

NA, Not available; HCC, Hepatocellular carcinoma; HR, Hazard ratio; CI, Confidence interval; OS, Overall Survival; PFS, Progress Free Survival.

### Published Articles Related to the Trials of Tivantinib in HCC

To identify the clinical studies investigating tivantinib in HCC, six databases, including MEDLINE, EMBASE, Cochrane Library, PsychINFO, SCOPUS, and ISI databases were systematically searched prior to May 1, 2021. Only studies reporting with the English language were considered to be eligible. The searching strategy in MEDLINE (PubMed) databases was: [(((“ARQ 197” (Supplementary Concept)] OR [ARQ197)) OR (ARQ-197)) OR (tivantinib)] AND [((((((((((((((((((“Carcinoma, Hepatocellular”(Mesh)] OR (Carcinomas, Hepatocellular)) OR (Hepatocellular Carcinomas)) OR (Liver Cell Carcinoma, Adult)) OR (Liver Cancer, Adult)) OR (Adult Liver Cancer)) OR (Adult Liver Cancers)) OR (Cancer, Adult Liver)) OR (Cancers, Adult Liver)) OR (Liver Cancers, Adult)) OR (Liver Cell Carcinoma)) OR (Carcinoma, Liver Cell)) OR (Carcinomas, Liver Cell)) OR (Cell Carcinoma, Liver)) OR (Cell Carcinomas, Liver)) OR (Liver Cell Carcinomas)) OR [Hepatocellular Carcinoma)) OR (Hepatoma)) OR (Hepatomas)].

After excluding duplicates, *in vitro* or *in vivo* studies, review articles, comments, case reports, and irrelevant articles, eight clinical studies (41, 42, 54–59) were finally included. The characteristics of the eight eligible studies were summarized in **Table 2**. These studies were published from 2013 to 2020 years. The study areas included Italy, Belgium, Germany, Canada, the USA, Australia, New Zealand, and Japan. Three clinical studies were phase I, two for phase II, and three for phase III. The cancer type was advanced HCC, unresectable HCC, and MET-high HCC. A total of 1,091 participants were included in the eight studies. The sample size ranged from 20 to 340 patients. The age of the patients ranged from 19-87 years. The methods of drug administration and dosage were orally 120/240/360 mg twice daily. In the biomarker analysis, MET overexpression was recorded at 34.6% to 100%.

**Table 2:** The characteristics of the 8 published phase 1/2/3 studies of tivantinib in HCC.

Study and references	Study area	Clinical phase	Cancer type, Number of patients	Age (years)	Therapies (Tivantinib)	Biomarker analysis (number of patients)	Therapeutic effects	Adverse events (%)
Santoro et al. (54)	Italy, Belgium, Germany, Canada, and USA	I	HCC, 21	47-80	360 mg, Orally, BID	NA	NA	11 patients (52%), including neutropenia, anemia, leucopaenia, etc.
Santoro et al. (42)	Italy, Belgium, Germany, Canada, and USA	II/Randomized	Advanced HCC, 107	27-85	240 mg *vs* 360 mg *vs* Placebo, Orally, BID	MET overexpression (34.6%)	Time to progression was longer for patients treated with tivantinib (1.6 months) than placebo (1.4 months); HR= 0.64, 90% CI: 0.43–0.94, *P*=0.04.	Neutropenia (14%)
Rimassa et al. (55)	International Multi-center Clinical Trial	III/Randomized	Advanced,MET-high HCC, 303	NA	120 mg *vs* Placebo, Orally, BID	MET overexpression (100%)	NA	NA
Puzanov et al. (56)	USA and Italy	I	Advanced HCC, 20	41-77	240 mg + sorafenib 400 mg, Orally, BID	MET-High (40%)	The overall response rate was 10%, the disease control rate was 65%. The median PFS was 3.5 months (95% CI: 3.0-11.1 months).	Rash (40%), diarrhea (38%), and anorexia (33%)
Okusaka et al. (57)	Japan	I	Advanced HCC, 28	Median: 65	120 mg *vs* 240 mg, Orally, BID	NA	NA	120 mg was considered tolerable, while 240 mg were associated with neutropenia or febrile neutropenia
Rimassa et al. (41)	Multi-center Clinical Trial	II/Randomized	HCC, 77	27-85	NA	MET-High (48%)	Survival in circulating MET-High patients was 7.0 months on tivantinib and 3.8 months on placebo, (HR 0.55, 95% CI, 0.28-1.06, *P*=0.07). The OS in circulating MET-Low patients was 7.5 months on tivantinib and 9.4 months on placebo, (HR 0.97, 95% CI, 0.51-1.85, *P*= 0.93)	NA
Rimassa et al. (58)	Australia, the Americas, Europe, and New Zealand	III/Randomized	Unresectable, progressed, or intolerant to sorafenib, 340	19-87	120 mg *vs* Placebo, Orally, BID	MET-High (53%)	Median overall survival was 8.4 months (95% CI 6.8–10.0) in the tivantinib group and 9.1 months (7.3–10.4) in the placebo group (HR=0.97, 95% CI: 0.75–1.25, *P*=0.81).	Ascites (7%), anaemia (5%), abdominal pain (4%), and neutropenia (4%).
Kudo et al. (59)	Japan	III/Randomized	MET-high HCC, 195	36-86	120 mg *vs* Placebo, Orally, BID	MET-High (52.3%)	Median PFS was 2.8 and 2.3 months in the tivantinib and placebo groups, respectively (HR= 0.74, 95% CI: 0.52-1.04, *P*= 0.082). Median OS was 10.3 and 8.5 months in the tivantinib and placebo group, respectively (HR= 0.82, 95% CI: 0.58-1.15, *P*>0.05).	Neutropenia (31.6%), leukocytopenia (24.8%), and anemia (12.0%)

NA, Not available; HCC, Hepatocellular carcinoma; HR, Hazard ratio; CI, Confidence interval; OS, Overall Survival; PFS, Progress Free Survival.

The therapeutic efficacies were inconsistent among the eight included studies. Santoro et al.’s study (phase 2) (42) have recruited 107 participants, they found that the time to progression was longer for patients treated with tivantinib (1.6 months) than those with placebo (1.4 months) (HR= 0.64, 90% CI: 0.43–0.94, *P*=0.04). In a small-sample study (phase 1) (56) developed by Puzanov et al., the authors observed that the overall response rate in patients who received 240 mg tivantinib plus sorafenib 400 mg was 10% and the disease control rate was 65%. In a multi-center clinical trial (phase 2) conducted by Rimassa et al. (41), it was shown that the survival in circulating MET-High patients was 7 months on tivantinib and 3.8 months on placebo (HR 0.55, 95% CI: 0.28-1.06, *P*=0.07). However, the OS in circulating MET-Low patients was not statistically significant between the study group and the control group, showing that the OS was 7.5 months on tivantinib and 9.4 months on placebo (HR 0.97, 95% CI: 0.51-1.85, *P*= 0.93). Afterward, the subsequent phase 3 trials (58) completed by Rimassa et al. continually showed that no significant differences were found between tivantinib and placebo in the OS (8.4 months *vs* 9.1 months, *P*=0.81). In a more recent study (phase 3) (59) conducted in Japan, Kudo et al. recruited 195 participants and suggested that there was no statistical significance between the tivantinib and the placebo in both the PFS and the OS (all *P*>0.05). The authors demonstrated that the median PFS was 2.8 and 2.3 months in the study group and the control, respectively (HR= 0.74, 95% CI: 0.52-1.04, *P*= 0.082). On the other hand, the median OS was recorded at 10.3 and 8.5 months in the tivantinib and placebo group, respectively (HR=0.82, 95% CI: 0.58-1.15, *P*>0.05).

In the safety analysis, most of the included studies indicated that neutropenia was the most common adverse event, the incidence rate ranged from 4% to 31.6%. Other frequent adverse events included anemia, ascites, abdominal pain, leucopenia, rash, diarrhea, and anorexia. In Okusaka et al.’ study (57), the authors pointed out that 120 mg of tivantinib was considered tolerable, while 240 mg were associated with neutropenia or febrile neutropenia.

## Discussion

Based on the current evidence, tivantinib, a tyrosine kinase inhibitor targeting the MET pathway, was widely studied in advanced MET-positive HCC, but no substantial benefit was proven. Only a phase 2 study developed by Santoro et al. (42) demonstrated that advanced HCC patients were benefited from tivantinib treatment. The authors observed that the median time to progression in patients with tivantinib was significantly longer than those who received a placebo (1.6 months *vs* 1.4 months, *P*=0.04). However, no significant differences were observed in the median PFS (tivantinib: 1.5 months, placebo: 1.4 months, *P*=0.06) and the median OS (tivantinib: 6.6 months, placebo: 6.2 months, *P*=0.63) between the two groups. Santoro et al. also found that the OS was slightly longer in patients who received 240 mg twice-daily dose than those with 360 mg twice-daily (7.5 months *vs* 6.4 months), but this difference was not significant. Results from two subsequent phase 3 studies (58, 59) with a large-sample size showed that tivantinib did not improve overall survival compared with placebo. The METIV-HCC study (58) revealed that advanced MET-high patients who received tivantinib 120 mg twice daily did not improve overall survival as compared to those with placebo. On the other hand, PFS was similar between the two groups. Subgroup analyses also indicated that no patient subgroups could benefit from tivantinib intervention. In line with the METIV-HCC study of Australia and Europe population, the Japanese Evaluation of Tivantinib in Hepatocellular Carcinoma (JET-HCC) study (59) also demonstrated that oral daily tivantinib did not significantly prolong the OS and the PFS in Japanese patients with MET-high HCC who had relapsed or were intolerant to sorafenib. Among the eight published phase 1/2/3 studies of tivantinib in advanced HCC, only one study (56) included tivantinib with other chemotherapeutics (i.e., sorafenib). This phase 1 trial without a placebo group showed that the overall response rate in advanced HCC patients was 10% and the disease control rate was 65% after 240 mg tivantinib combined with sorafenib 400 mg. The authors concluded that combining the VEGF inhibitor (i.e, sorafenib) and the selective MET inhibitor (i.e, tivantinib) might provide synergistic or additive anti-tumor activity overcoming the resistance to sorafenib in treating advanced solid tumors, including HCC. Though the outcomes were exhilarating, no phase 2 or phase 3 trials of tivantinib plus sorafenib in advanced HCC are ongoing. Cabozantinib, an inhibitor targeting VEGF, MET, and the “anexelekto” receptor tyrosine kinase, was approved by FDA in 2012 to treat metastatic medullary thyroid cancer (56). Based on the promising survival outcomes of the randomized, placebo-controlled, phase 3 CELESTIAL trial in patients with advanced HCC, cabozantinib was approved for the treatment of unresectable and progressing HCC after the failure of sorafenib in Europe (2018) and in the USA (2019) (60, 61). Therefore, dual concomitant inhibition of MET and VEGF pathways may be an effective strategy to treat advanced and progressive HCC.

There are many postulated causes for this inconsistency between the phase 2 (ARQ 197-215) and phase 3 trials (METIV-HCC). As reported in the METIV-HCC studies (58), several possible reasons might respond for the difference, (i) the sample size in the phase 2 study is small (107 participants), which might cause introduction or selection bias; (ii) the formulation of tivantinib was different (phase 2 study: capsule, phase 3 study: tablet), which might disturb the drug absorption or elimination; (iii) laboratory that assessed the MET expression was different in the two studies; (iv) the number of biopsies obtained before and after sorafenib treatment; (v) the number of patients with MET-high tumors identified before and after sorafenib therapy; (vi) exclusion of patients with pleural effusion in the phase 3 study, which might be the most important reason; (vii) in the phase 3 study, only the patients with the biopsy results could be recruited, resulting in less-aggressive disease were included and those with disease progression were excluded. Weekes et al. (45) also pointed out that some biases in the phase 2 study should be noted due to the sample size was small and the subset analysis might be not representative. Meanwhile, due to the intratumoral molecular heterogeneity and variability of intratumoral MET expression in HCC, it is still not clear to judge whether the HCC patients are sensitive to MET inhibition by using an immunohistochemical score of 2 for MET expression. The prognostic value of MET level assessed by immunohistochemistry on tumors after sorafenib treatment remains to be clearly defined.

Different formulations and dosing of tivantinib in the phase 2 study and phase 3 study need to be further discussed. Rimassa et al. (58) demonstrated that the dosing of tivantinib changes from 360/240 mg twice daily to 120 mg twice daily in order to reduce the incidence of grade 3 or worse neutropenia. Though the authors suggested that the pharmacokinetics of tivantinib administrated with 120 mg tablets and 240 mg capsules were similar, no data were shown on the MET activity before and after tivantinib treatment. Therefore, it is uncertain whether the therapeutic effects of tivantinib or the inhibitory efficiency on MET activity are equivalent under 120 mg tablets and 240 mg capsules treatment. Another concern is that there were two dosings of tivantinib in the phase 2 study, 240 mg and 360 mg twice daily. We could find that 54% (38/71) of the participants in the phase 2 study (42) have received the 360 mg twice daily dose. However, there is only one dose of 120 mg tablets twice daily in the phase 3 study, irrespective of whether 120 mg tablets and 240 mg capsules are equivalent. However, the lower dose of tivantinib in the phase 3 study (58) is unavoidable due to the serious adverse events. Based on the above evidence, we should note that the different formulations and dosing of tivantinib might play roles in the inconsistent results of the phase 2 study and phase 3 study.

Similar to the multicentric METIV-HCC study, the JET-HCC study (59) in Japan also failed to meet the primary endpoints of OS and PFS under tivantinib treatment. Kudo and their colleagues are the investigators of the JET-HCC study (59), they emphasize the difference in the enrolled participants between the phase 2 and phase 3 studies. In the inclusion criteria in the two trials, the enrolled HCC patients were irrespective of c-Met expression and were retrospectively analyzed MET high or MET-low in the phase 2 study (ARQ 197-215), while the participants in the phase 3 study (METIV-HCC) have confirmed the MET-high levels during the screening test. Due to this study designed, Kudo et al. also pointed out that the duration of the recruitments was longer in the phase 3 study than the phase 2 study, resulting in a bias as patients with early progress during the screening period might have dropped out of the enrollment. On the other hand, the patients in the phase 3 study were confirmed to MET-high expression, thus early progressions might occur in some of the patients due to MET-high is a poor prognostic factor for HCC. Therefore, differences in study design may be one of the important factors for the inconsistencies between ARQ 197-215 and the METIV-HCC study.

As for the safety analyses, the adverse events of tivantinib treatment were similar to among the I/II/III clinical trials regardless of study areas. Hematological toxicities were the common adverse events under tivantinib interventions. In the METIV-HCC study (58), the most frequent grade 3 or worse treatment-related adverse events of tivantinib were ascites, anemia, abdominal pain, and neutropenia. This study also indicated that tivantinib 240 mg twice daily dose was poorly tolerated in the tablet formulation, while treatment-emergent adverse events could be manageable at 120 mg. In the JET-HCC study, it was reported that the frequency and severity of tivantinib-mediated hematotoxicity were similar between CYP2C19 extensive metabolizers and poor metabolizers (57). This study also showed that neutropenia at any grade and that at grades ≥3 were 43.6% and 31.6%, which was remarkably higher than that of the METIV-HCC study (any grade: 8%, grades ≥3: 4%) even under the same dose of 120 mg twice daily. It is known that the bodyweight of the Asian population is generally less than the Western population. Therefore, one of the explanations for the higher rate of neutropenia in the JET-HCC study might be due to the plasma concentration of tivantinib was higher in the Japanese sample than that of the Western population under the same dosing level (59). On the other hand, it can be speculated that a high concentration of tivantinib may have a superior antitumous effect on HCC. However, neither the JET-HCC nor the METIV-HCC study has been proved to prolong the PFS and OS in patients under tivantinib treatment. As reported by the same research team, the safety analyses from phase 2 (42) and the phase 3 study (58) demonstrated that a higher rate of neutropenia was observed in a 360 mg dose of tivantinib than 120 mg dose (14% *vs* 4%).

Based on the above evidence, advanced HCC patients do not benefit from tivantinib therapy alone. So, does this mean that MET (c-Met) inhibition may not be an appropriate choice for patients with advanced HCC? The answer appears to be indeterminate. Cabozantinib, a multi-kinase inhibitor against VEGF receptors 1-3, c-Met, and the TAM receptors (TYRO3, AXL, MER) family, has been approved for advanced HCC patients who are resistant to sorafenib (62). In phase 3 CELESTIAL study, advanced HCC patients with cabozantinib treatment have gained a significant benefit on the OS and PFS (60). Since MET is one of the direct targets for cabozantinib, MET inhibitory activity may be therefore partially responsible for the therapeutic effects of cabozantinib in treating progressing HCC patients who were previously treated with sorafenib. Atezolizumab (a programmed death-ligand 1, PD-L1 inhibitor) combined with bevacizumab (a monoclonal antibody that targets vascular endothelial growth factor, VEGF) have been reported to significantly prolong the overall and progression-free survival outcomes than sorafenib in patients with unresectable HCC (63). Interestingly, it is reported that MET inhibition could block the expression of PD-L1 on tumor cells (64). In addition, a recent *in vivo* study has shown that MET inhibitors combined with anti-PD1 and anti-PD-L1 produced additive effects on inhibiting the growth of HCCs in mice (65). On the other hand, studies have shown that MET signaling closely interacts with the VEGF and VEGF receptor (VEGFR) pathway (66). This assumption might be supported by the observation that MET activation elevates the VEGF expression and angiogenesis (67). Therefore, whether the blockage of the MET signaling involves the therapeutic action of atezolizumab plus bevacizumab for patients with advanced unresectable HCC needs further investigations.

Since an emerging role of precision and personalized medicine for cancer treatment, c-Met or MET serving as a biomarker for the management of cancer merits further exploration. Based on numerous previous reports, MET amplification, overexpression is considered to be an effective predictor for early diagnosis and prognostic outcomes in patients with multiple types of malignancies (68, 69). Besides, MET variation has also been identified to serve as a predictive biomarker of the response to treatment with MET-targeted therapies, either monotherapy or combination therapy (70, 71). However, the predictive value of MET-related biomarkers as useful tools to screen patients for c-Met targeted therapies is still controversial. The possible explanations for this debate may be correlated to the two main facts. First, it was reported that only MET-addicted tumors might respond to MET-targeted agents (72). Second, the assessments of MET expression are lack standardization of methodologies (i.e., different antibodies and staining protocols) and scoring rules (i.e., the cut-off points) (68, 73). The above evidence may explain the discordant findings between MET-high and MET-low, as well as the phase 2 and phase 3 trial of tivantinib in treating advanced HCC. The randomized phase 2 study showed a significant time to tumor progression (TTP), PFS, and OS benefit from tivantinib treatment in MET-high patients (42). But, among the patients with MET-low expression, the survival outcomes were not statistically significant between tivantinib and placebo, indicating that c-Met expression might be predictive of the response to tivantinib treatment (42). Furthermore, two subsequent phase 3 trials revealed that tivantinib could not significantly improve the OS as compared to the placebo group, in treating MET-high advanced HCC (58, 59). As aforementioned, these inconsistent results might be due to the detecting methodologies and the definitions of MET-high were different among these phase 2 and phase 3 studies. Therefore, future precisely incorporating biomarker analyses and HCC molecular subclasses may help to screen those patients who may benefit from c-Met inhibitors (i.e., tivantinib) treatment. After enriching by prospectively genetic and pharmacological testing, we believe that the combination of HGF/MET-targeted agents (i.e., tivantinib) with conventional chemotherapeutics or molecularly targeted agents (i.e., EGFR, VEGFR, and PI3K/Akt targeting agents) may provide the optimal personalized treatment regimens for advanced HCC patients.

### Contribution to the Field

The strengths of the present review study include multiple aspects. First, this study has summarized all the clinical evidence of the potent antitumor activity of tivantinib in advanced HCC. We included and analyzed not just restricted to the published data but also the clinical trials recorded in the ClinicalTrials.gov, even though some of which have resulted in failure. Previously, there are several review articles (12, 74, 75) reported with the narrow topic of tivantinib in treating advanced HCC, which were published ranged from 2013 to 2017. There are two important phase 3 trials (58, 59) published with the outcomes after the year 2017, but no review studies have summarized the up-to-date evidence. As a result, we have conducted the current review study which can provide comprehensive relevant information about the exact therapeutic effects of tivantinib on advanced HCC. In addition, we have shown that the most contrasting and detailed data among different phase 2 and phase 3 trials. Second, we have described an original schematic diagram of the molecular mechanisms of tivantinib in treating HCC. Currently, controversy does exist regarding the mechanism of action of tivantinib and whether a selective c-MET inhibition is the major anti-tumor effect of the drug. As shown in **Figure 1**, tivantinib exerts its antitumor activity mainly through two major modes, including inhibition of c-MET and dysregulation of microtubules. In the action of selective inhibition of c-MET, tivantinib exhibits an antitumor effect by inhibiting MET autoactivation by stabilizing the inactive nonphosphorylated configuration of the kinase, resulting in preventing of the downstream signaling pathways. In the action of disruption microtubule function, tivantinib exerts the antitumor activities by microtubule depolymerization, resulting in G2/M arrest and the blockage of cell mitosis, thus promotes apoptosis.

### Perspectives of c-Met Inhibitor

According to the research results from phase 3 studies, tivantinib was shown to fail in treating advanced HCC. As reported, monotherapy of targeting c-MET has failed to exert significant clinical efficacy in the majority of malignancies (43). However, some c-MET inhibitors have been proved to serve as an effective therapeutic option for certain cancers. For example, crizotinib, a selective c-MET inhibitor, was approved for marketing by the US FDA for patients with ALK mutation-positive non-small-cell lung cancer (NSCLC) (76). Cabozantinib was approved for marketing by FDA in treating medullary thyroid cancer (77). Capmatinib was confirmed to be effective for treating multiple cancers, such as patients with c-MET-dysregulated advanced solid tumors, glioblastoma, HCC, NSCLC, and colorectal cancer (43). On the other hand, monotherapy of targeting c-MET may be of no clinical importance, but the combinations of c-MET-targeted treatment have tremendous therapeutic potential for cancers. Crizotinib, a small-molecule tyrosine kinase inhibitor of MET, exhibited a significant anti-tumor effect in breast cancer cells when combined with endocrine drugs (78). Imura et al. (79) demonstrated that combined targeting of mTOR and c-MET signaling pathways might be an effective management of epithelioid sarcoma. Xu et al. (80) indicated that combined EGFR/MET inhibition is effective in treating lung cancer. As for tivantinib, it was reported that erlotinib plus tivantinib showed improved efficacy over erlotinib monotherapy in NSCLC, resulting in significantly increased PFS (81, 82). However, some investigators also found that combined tivantinib plus cetuximab did not significantly improve the survival compared with cetuximab alone in an unselected head and neck squamous cell carcinomas population (83). In the future, tivantinib or c-MET-targeted therapy in cancers, either monotherapy or combinations, needs further investigations.

## Conclusions

In summary, results from the two phase 3 studies (METIV-HCC and JET-HCC) indicated that tivantinib was not associated with significantly better OS and PFS outcomes than the placebo in patients with advanced unresectable HCC. Several factors may contribute to the inconsistency between the phase 2 and phase 3 studies of tivantinib, including the sample size, drug dosing, study design, and the rate of MET-High. Some positive studies of the novel drugs (i.e., cabozantinib and atezolizumab) reported with a significant efficacy on patients with progressive HCC indicate that MET signaling may involve in the underlying mechanisms for the therapeutical effects exerted by these drugs. High selective MET inhibitors combined with a biomarker-driven patient selection may provide a potentially viable therapeutic strategy for patients with advanced unresectable HCC.

## Author Contributions

SZ, KW, LW, and WW contributed to conceive and design the study. HJ, LM, and CP performed the article searching. KW and CJ extracted the data. LW and SZ wrote the manuscript. KW, JM and SZ supervised the manuscript. All authors contributed to the article and approved the submitted version.

## Funding

This work was supported by the grants from Science and Technology Planning Project of Taizhou City, Zhejiang Province (ID: 20ywb40); the High-level Hospital Construction Research Project of Maoming People’s Hospital; the Zhejiang Province Public Welfare Technology Application Research Project (No. LGF21H160022), Taizhou Social Development Science and Technology Plan Project (No. 21ywb26 and 21ywb29), Zhejiang Medical and Health Science and Technology Program (No. 2017KY711 and No. 2022RC297) and Natural Science Foundation of Zhejiang Province (No. Q22H044253).

## Conflict of Interest

The authors declare that the research was conducted in the absence of any commercial or financial relationships that could be construed as a potential conflict of interest.

## Publisher’s Note

All claims expressed in this article are solely those of the authors and do not necessarily represent those of their affiliated organizations, or those of the publisher, the editors and the reviewers. Any product that may be evaluated in this article, or claim that may be made by its manufacturer, is not guaranteed or endorsed by the publisher.

## Abbreviations

CI, Confidence interval; NA, Not available; HBV, Hepatitis B virus; HCC, Hepatocellular carcinoma; HGF, Hepatocyte growth factor; HR, Hazard ratio; c-Met, Cellular mesenchymal-epithelial transcription factor; OS, Overall Survival; PFS, Progress Free Survival; SF, Scatter factor; TACE, Transarterial chemoembolization
